# Exploring Motivation and Barriers to Physical Activity Among Educated Adult Saudi Women at Taif University

**DOI:** 10.1186/s13102-024-01030-0

**Published:** 2024-12-20

**Authors:** Sarah M. Alajlan, Obaidalah H. Aljohani, Wissal Boughattas

**Affiliations:** 1 The Department of Leadership and Educational Policies, Taif University, Taif, Saudi Arabia; 2 The Department of Sport Sciences and Physical Activity, Taif University, Taif, Saudi Arabia

**Keywords:** Motivation and barriers, Physical activity, Self-determination theory, And adults Saudi women

## Abstract

Although physical activity (PA) offers numerous mental and physical health benefits, adult Saudi women face unique social and cultural barriers to participating in PA. This study aimed to explore the motivational factors and obstacles influencing PA participation among adult Saudi women at Taif University. Using Self-Determination Theory (SDT) as the framework, a questionnaire was developed and administered to 706 participants, including students, administrators, and faculty members. Results indicated that psychological motivations were the strongest drivers of PA engagement, while physical and service-related limitations were the primary barriers to regular participation. Notably, women aged 30 to 40 showed a greater inclination toward regular physical activity than other age groups. The results indicate a requirement for focused initiatives and enhancements in infrastructure to overcome obstacles and promote physical activity engagement among women in Saudi Arabia. Further research is needed to validate these findings and to inform strategies for promoting PA among Saudi women.

**Supplementary Information:**

The online version contains supplementary material available at 10.1186/s13102-024-01030-0.

## Background

The substantial advantages of physical activity (PA) for mental and physical health are well known. However, many women worldwide, particularly in Saudi Arabia, have additional obstacles that prevent them from regularly participating in PA. Cultural, social, and structural elements frequently combine to cause these difficulties [2]. As part of a larger effort to enhance public health, Saudi Vision 2030 emphasizes the significance of raising PA among all residents, with a particular focus on female engagement [25]. Despite these initiatives, Saudi women’s involvement rates are still low because of barriers such restricted access to facilities, a lack of social support, and ingrained cultural norms that dissuade women from participating in physical activities in public [2, 4, 27]. Restricted access to sports facilities remains a significant challenge for many women, particularly in smaller cities and rural areas [8, 12]. Additionally, cultural expectations and societal norms continue to play a pivotal role in limiting women’s participation, as many feel discouraged from engaging in physical activities in public spaces [13].

Historically, women in Saudi Arabia had limited exposure to physical education in schools or universities and minimal awareness of PA’s health benefits. Funding and support for women’s sports facilities also remain significantly lower than for men’s, reinforcing traditional norms and creating structural barriers to participation [7]. In response, the Ministry of Education introduced physical education programs for girls in public schools in 2017, and several universities began offering PA and sports science programs specifically for women. Under Saudi Vision 2030, the establishment of women-only gyms aims to provide safe and accessible spaces to encourage female participation in sports [20].

Despite these initiatives, studies indicate that Saudi women are still three times less active than men, with participation motivations primarily related to health maintenance, weight management, and social connection. Common barriers include lack of time, self-confidence, and access to facilities [3, 5]. This study, conducted at Taif University, seeks to explore Saudi women’s perceptions of their motivations for PA and the barriers they face. Specifically, it examines psychological, fitness-related, health-related, athletic inclination, and social motives, as well as barriers associated with physical and service limitations, professional or educational challenges, societal and family expectations, psychological obstacles, lack of awareness, and health-related concerns.

### Theoretical framework

Self-Determination Theory (SDT) serves as the primary theoretical framework for this study, as it provides a comprehensive model for understanding motivation through three core psychological needs: autonomy, competence, and relatedness. According to SDT, individuals experience intrinsic motivation when these needs are met, which leads to higher engagement in activities that contribute to personal growth and well-being (14,15). SDT distinguishes between intrinsic motivation, where individuals participate in activities out of personal satisfaction, and extrinsic motivation, which is driven by external rewards or pressures [22]. In the context of PA, SDT suggests that individuals who experience intrinsic satisfaction from exercise are more likely to maintain their participation long-term, whereas those motivated by external factors may struggle to sustain involvement [23].

While intrinsic motivation is central to long-term engagement, extrinsic factors, such as social pressures or external rewards, can play a significant role in initiating participation, particularly in cultural contexts where external influences strongly shape behavior. Research highlights how external motivators can shift along a continuum toward more self-determined forms of motivation when aligned with personal values and goals, ultimately contributing to sustained behavior. This perspective enriches the understanding of Saudi women’s participation in PA, as it considers the interplay between intrinsic satisfaction and the influence of culturally specific external factors [17].

### Study objectives

The purpose of this study is to investigate the barriers and motivating factors influencing adult Saudi women’s participation in physical activity at Taif University. In particular, it looks into the impact of:

Motivating Elements: This entails looking at important motivators like psychological ones (stress relief, self-confidence), fitness-related ones (physical condition improvement), health-related ones (health management and disease prevention), athletic inclinations (sport interest), and social ones (peer interaction).

Barriers: The study tackles issues with psychological barriers (self-doubt, fear of judgment), professional or educational demands, societal norms and family expectations, physical and service limitations (availability of facilities and services), and health-related concerns that may hinder participation.

These objectives align with the Self-Determination Theory (SDT) framework to provide insights into how intrinsic and extrinsic motivational factors, as well as perceived barriers, impact PA engagement among Saudi women in a higher education context. This alignment is intended to facilitate understanding of the unique social, cultural, and structural factors that affect PA participation among this population, ultimately guiding effective strategies to improve PA engagement in line with Saudi Vision 2030 [25].

## Methods

### Study population and sample

The study targeted female learners, administrators, and faculty members at Taif University. The total population included 2,762 adult females, consisting of 437 administrators, 1,100 graduate students, and 1,225 faculty members. After distributing the survey and applying, a final sample size of 706 participants was obtained, including 95 administrators, 300 graduate students, and 311 faculty members. This final sample size will be clarified in Table 1.

**Table 1:** Demographic data

Category	Subcategory	*N* %
Age (years)	Less than 30	280 39.7%
	30–40	322 45.6%
	> 40	104 14.7%
Position	Administrator	95 13.5%
	Graduate student	300 42.5%
	Faculty member	311 44.1%
Marital status	Single	302 42.8%
	Married	366 51.8%
	Divorced	38 5.4%
Total		706 100.0%

### Study design

This study is a descriptive, exploratory quantitative research that employs a self-developed questionnaire to explore the motivations and barriers to physical activity among adult Saudi women at Taif University. The instrument was designed through a thorough review of relevant literature on the topic of motivations and barriers to physical activity.

### Data collection

Data were collected through an online structured questionnaire distributed via email and the university’s internal communication system. The questionnaire was divided into three sections: demographic information, motivations for PA, and barriers to PA.

### Statistical analysis

The statistical analysis for this study included both descriptive and inferential statistics. Descriptive statistics, such as means, standard deviations, and percentages, were used to summarize demographic data and key study variables. For inferential analysis, a four-way ANOVA was conducted to assess differences in motivation and barriers to physical activity based on age, position, marital status, and current engagement in physical activity or sports. Significant differences emerged for age and position variables, though marital status showed no significant effect. To explore these differences further, Scheffé post-hoc comparisons were applied, revealing that women over 40 and administrators reported facing more barriers in engaging in physical activity.​.

### Ethical considerations

This study was approved by the Research Ethics Committee at Taif University, adhering to the university’s Bioethics Code (approval number 43–072), ensuring compliance with ethical standards for human subject research.

### Survey instrument

The survey instrument was a questionnaire initially developed in English, then translated into Arabic by expert translators to ensure accuracy and comparability between the two versions. The questionnaire was divided into three sections: the first gathered basic demographic information (age, position, and marital status). The second section focused on motivations for physical activity (PA), comprising five subcategories: social, psychological, health, fitness-related, and athletic-inclination motives. The third section addressed barriers to PA and included six categories: professional or educational barriers, physical and service challenges, health challenges, psychological challenges, societal/family challenges, and lack of awareness. The five-point scale was as follows: 1 = strongly disagree, 2 = disagree, 3 = undecided, 4 = agree and 5 = strongly agree. The English items for motivation and challenge scales are provided in Appendix A.

### Reliability and validity

The questionnaire items were reviewed by a panel of fourteen experts in sports and PA to assess clarity, relevance, and alignment with each axis. The experts provided feedback, leading to revisions to enhance item clarity and ensure that they accurately represented each axis. Following these modifications, a pilot study with 35 participants, similar to the main study population, was conducted to assess clarity and comprehension. Correlation coefficients for motivators of PA ranged from 0.36 to 0.92 for item-to-total questionnaire correlations and 0.39–0.95 for item-to-axis correlations. For barriers to PA, item-to-total correlations ranged from 0.37 to 0.73, and item-to-axis correlations were between 0.45 and 0.91, all within acceptable levels, so no items were removed.

Reliability was further tested using the test-retest method on an exploratory sample with a three-week interval. Pearson correlation coefficients ranged from 0.90 to 0.93 for motivators and 0.91–0.93 for barriers, demonstrating high stability. Additionally, Cronbach’s alpha was calculated to confirm internal consistency, yielding alpha coefficients of 0.76–0.91 for motivators and 0.78–0.95 for barriers, indicating a reliable instrument.

## Results

Research question 1: What motivates adult Saudi women to participate in physical activity (PA) at Taif University? To answer this question, the means and standard deviations (SD) for each motivational factor influencing Saudi women at Taif University to engage in PA were calculated. Psychological motives ranked the highest, with a mean (M) of 4.37 (SD = 0.678), indicating their strong influence. Fitness-related motives followed, with a mean of 4.19 (SD = 0.654), while social motives were ranked the lowest, with a mean of 3.50 (SD = 0.707). The overall mean for motivational factors was 4.04 (SD = 0.503), indicating a generally high level of motivation among participants. Table 2 provides a summary of the means and standard deviations for each motivational factor:

**Table 2:** Mean and standard deviation for the five motivation factors for PA

Motivation for PA	Mean (M)	SD	Rank
Psychological motives	4.37	0.678	1
Fitness-related motives	4.19	0.654	2
Health motives	4.07	0.563	3
Athletic-inclination motives	4.06	0.789	4
Social motives	3.50	0.707	5
Total score	4.04	0.503	

Research question 2: What barriers are facing adult Saudi women who wish to engage in PA at Taif University? To answer the question, means and standard deviations were determined. The highest-ranked items relating to the barriers were physical and service obstacles. The mean for this axis was 3.55 (SD = 0.691). Professional or educational barriers were the second most important obstacle to engagement in PA, with a mean of 2.74 (SD = 0.977). Health challenges ranked last, with a mean of 1.93 (SD = 0.676). The overall mean for the challenges scale was 2.65 (SD = 0.529), indicating moderate challenges among the participants. As seen in Table 3, the means ranged between 1.93 and 3.55.

**Table 3:** Mean and standard deviation for the six barriers factor to PA

Challenges for PA	Mean (M)	SD	Level
Physical and service challenges	3.55	0.691	1
Professional or education challenges	2.74	0.977	2
Societal/family challenges	2.66	0.826	3
Psychological challenges	2.23	0.886	4
Lack of awareness	2.12	0.882	5
Health challenges	1.93	0.676	6
Total score	2.65	0.529	

Research question 3: Is there a statistically significant difference, based on age, position, and marital status, in motivation to participate in physical activity (PA) and the challenges facing Saudi women at Taif University? To address this question, means (M) and standard deviations (SD) for each demographic category (age, position, and marital status) were calculated to identify variations in motivation levels. The findings are summarized in Table 4.

**Table 4:** Motivation to participate in PA by age, position, and marital status

Factor Group	Group	Mean(M) SD	*N*
Age (years)	Less than 30	3.92 0.554	280
	30–40	4.14 0.441	322
	> 40	4.07 0.472	104
Position	Administrator	3.95 0.352	95
	Graduate student	4.08 0.516	300
	Faculty member	4.03 0.527	311
Marital status	Single	3.97 0.562	302
	Married	4.10 0.449	366
	Divorced	4.10 0.450	38

The data indicate variations in motivation levels across different demographic groups. Participants aged 30–40 years reported the highest motivation to participate in PA (M = 4.14, SD = 0.441), while those under 30 years had slightly lower motivation (M = 3.92, SD = 0.554). Among positions, graduate students exhibited the highest motivation (M = 4.08, SD = 0.516), followed by faculty members (M = 4.03, SD = 0.527), with administrators showing the lowest motivation (M = 3.95, SD = 0.352). Regarding marital status, both married and divorced participants had similar motivation levels (M = 4.10), which were slightly higher than those of single participants (M = 3.97).

To determine if these differences were statistically significant, a four-way Analysis of Variance (ANOVA) was performed, considering age, position, marital status, and current engagement in PA or sport as independent variables. The ANOVA results are summarized in Table 5.

**Table 5:** Four-way ANOVA result

Source	Sum of Squares	df	Mean Square	F	Sig. (*p*-value)
Age	7.984	2	3.992	17.572	0.000**
Position	6.749	2	3.374	14.853	0.000**
Marital Status	0.309	2	0.154	0.68	0.507
Current Engagement in PA	6.034	1	6.034	26.562	0.000**
Error	158.58	698	0.227		
Total	178.72	705			

Note *p* < 0.05

The ANOVA results revealed that: Age had a statistically significant effect on motivation to participate in PA (F (2, 698) = 17.572, *p* = 0.000). Position also showed a significant effect (F(2, 698) = 14.853, *p* = 0.000). Marital status did not have a significant effect (F(2, 698) = 0.680, *p* = 0.507) Current engagement in PA or sport was significant, with those not currently engaged showing higher motivation (F(1, 698) = 26.562, *p* = 0.000).

Scheffé post-hoc comparisons were conducted to clarify significant differences in motivation scores between age and position groups. The analysis revealed significant differences at the 0.05 level between women under 30 and those in the 30–40 and over 40 age groups, with both older groups showing higher motivation levels. This suggests that women aged 30 and above are generally more motivated to engage in physical activity than the younger counterparts.

For the position variable, Scheffé post-hoc comparisons indicated significant differences specifically between administrators and graduate students, with graduate students reporting higher motivation. This finding may suggest that graduate students have a greater interest in or incentive for physical activity compared to administrators. The results for the Scheffé post-hoc comparisons are reported in Table 6.

**Table 6:** Post-hoc comparison of results by age and position for the motivation axis

		Mean	Less than 30	30–40	>40
Age (years)	Less than 30	3.92			
	30–40	4.14	0.23*		
	> 40	4.07	0.15*	0.08	
		Mean	Admin	GS	Faculty
Position	Administrator (Admin)	3.95			
	Graduate student (GS)	4.08	0.14*		
	Faculty member (Faculty)	4.03	0.08	0.05	

Note*p < 0

For the barriers to engaging in PA, mean and standard deviation, based on age, position, marital status and currently engaging in PA are reported in Table 7.

**Table 7:** Mean and standard deviation for barriers to participation in PA

Factor Group		Mean(M) SD	*N*
Age (years)	Less than 30	2.64 0.474	280
	30–40	2.58 0.581	322
	> 40	2.90 0.425	104
Position	Administrator	2.80 0.452	95
	Graduate student	2.59 0.528	300
	Faculty member	2.66 0.543	311
Marital status	Single	2.58 0.500	302
	Married	2.72 0.552	366
	Divorced	2.56 0.450	38
Engage in	Yes	2.48 0.528	340
PA	No	2.81 0.478	366

The four-way ANOVA analysis showed no significant differences at the 0.05 level for marital status (F = 1.239, *p* = 0.290). However, a statistically significant difference was found for the variable regarding current engagement in PA or sport, with an F-value of 74.000 and a significance of 0.000. Table 8 shows significant differences based on age, position, and engagement in PA.

**Table 8:** Four-way ANOVA result

Source	Sum of Squares	df	Mean Square	F	Sig. (*p*-value)
Age	6.528	2	3.264	13.635	0.000**
Position	3.219	2	1.61	6.724	0.000**
Marital Status	0.593	2	0.297	1.239	0.29
Current Engagement in PA	17.714	1	17.714	74	0.000**
Error	167.09	698	0.239		
Total	197.55	705			

Note: *p* < 0.05

The ANOVA results revealed significant differences at the 0.05 level for the age and position variables. For the age variable, the F-value was 13.635, with a significance of 0.000. For the position variable, the F-value was 6.724, with a statistical significance of 0.001. To further explore these differences, Scheffé post-hoc comparisons were used. The post-hoc analysis indicated significant differences at the 0.05 level between women over 40 and those in the under-30 and 30–40 age groups, with the over-40 group facing more challenges. For the position variable, the Scheffé post-hoc comparisons showed significant differences between administrators and graduate students, with administrators facing greater challenges. The results of the Scheffé post-hoc comparisons are reported in Table 9.

**Table 9:** Post-hoc comparison of the results by age and position for the barriers

		Mean	Less than 30	30–40	> 40
Age (years)	Less than 30	2.64			
	30–40	2.58	0.05		
	> 40	2.90	0.26*	0.31*	
		Mean	Admin	GS	Faculty
work	Administrators (Admin)	2.80			
	Graduate students (GS)	2.59	0.22*		
	Faculty members (Faculty)	2.66	0.14	0.08	

*Note: *p* < 0.05

## Discussion and conclusion

This study, conducted at Taif University, aimed to examine Saudi women’s motivations for engaging in regular physical activity (PA) and the barriers they encounter. The findings revealed that the most cited motivations were psychological satisfaction and fitness, aligning with [18], who identified intrapersonal, psychological, and material factors as key motivators. Additionally, the results are consistent with [1], who reported that fitness, health, and psychological well-being were common motivators for both men and women (19) highlighted the importance of internal motivation for sustained PA engagement, which this study also observed, as participants with intrinsic motivations tended to engage more consistently [21]. support this, emphasizing that intrinsically motivated individuals engage in activities for enjoyment, which encourages long-term participation.

In contrast, individuals driven by extrinsic factors, such as external rewards or pressures, often exhibit less sustained engagement [23]. Although external motivators may initially prompt PA engagement, intrinsic motivations generally develop over time, leading to a more enduring habit [23]. These results support Self-Determination Theory, specifically emphasizing the importance of feeling competent, as individuals who showed higher levels of psychological satisfaction and fitness-related motivation were more likely to be involved in physical activity. Nevertheless, the obstacles identified, like restricted entry to resources, pose difficulties in meeting the need for independence, impacting ongoing involvement.

Health, athletic inclination, and social motives were cited less frequently than psychological and fitness factors. This finding differs from prior Saudi studies indicating that weight loss and health were primary reasons for PA [3, 5]. Such differences might be due to the demographic and cultural uniqueness of Taif, a growing city with different social dynamics from major urban centers like Riyadh or Jeddah. Additionally, the study participants, being affiliated with a single institution, might have shared similar sociocultural influences, unlike other studies with more diverse samples.

Regarding barriers, physical and service-related obstacles were the primary constraints, a finding consistent with previous studies [6, 24]. Participants reported limited access to public facilities, structured programs, and sports clubs, restricting their ability to engage in daily PA. Enhancing infrastructure in smaller cities and raising awareness about physical activity could help reduce these obstacles, in line with the objectives of Saudi Vision 2030. These findings are consistent with previous research that highlighted similar barriers among women in Saudi Arabia [9, 10]. In contrast, other Saudi studies [3, 5, 12] have identified lack of knowledge, time, motivation, and self-confidence as significant barriers. Further research with larger and more diverse samples could help confirm these results across different regions in Saudi Arabia.

The third research question addressed whether demographic variables affect motivation for PA. The results indicated that women aged 30–40 were more motivated than other age groups, suggesting that women in this age range, often having completed their education and entering the workforce, may find PA to be more essential. Furthermore, women over 40 who held administrative positions appeared to encounter fewer constraints. These results are in line with [26], who identified age and educational level as factors in PA motivation [26]. also found that marital status correlated with PA practices, with married individuals demonstrating less motivation, a trend that may also apply to Saudi cultural contexts [11].

This study highlights the nuanced effects of sociodemographic factors on PA motivation and barriers. Age and job position, in particular, were determinants of PA practices among participants, underscoring the need for targeted strategies to support PA across different demographic groups. Depending on their roles and responsibilities, professional groups display varying degrees of physical activity. For instance, aspiring educators exhibit a range of physical activity levels, which frequently corresponds with their understanding of healthy lifestyle choices and the demands of their line of work. In order to improve these groups’ well-being and increase their efficacy in future professional roles, it is imperative that physical activity be encouraged [16]. Future research should investigate these variables in larger, more representative Saudi samples, including various regions and demographic categories (e.g., gender, marital status, and student versus employee).

The findings of this study underscore the significance of psychological and fitness-related motivations in driving Saudi women’s participation in PA. Conversely, physical and service-related challenges present significant barriers. These results align with previous research, highlighting the need for targeted interventions and improvements in infrastructure to better support women’s engagement in PA.

In conclusion, the study found that psychological motivation and fitness goals were primary motivators for PA among adult women at Taif University. Age and job position also played roles in motivation levels, while the main barriers to PA were physical and service-related obstacles. This study underscores a lack of previous research exploring the interaction between motivations and barriers in PA. Further empirical studies are essential to expand on these findings, offering insights into how motives and barriers interact and affect PA practices within diverse Saudi populations.

## Electronic supplementary material

Below is the link to the electronic supplementary material.

Supplementary Material 1

## Data Availability

The datasets used and/or analysed during the current study are available from the corresponding author on reasonable request.
