# Supplementary material for: Exploring Motivation and Barriers to Physical Activity Among Educated Adult Saudi Women at Taif University
Source: BMC Sports Sci Med Rehabil. 2024 Dec 20;16:249. doi: 10.1186/s13102-024-01030-0 (PMC11660610; doi:10.1186/s13102-024-01030-0)
Supplement: Supplementary file 1 — Supplementary Material 1 [file 13102_2024_1030_MOESM1_ESM.pdf]

A copy of the survey.

Appendix A

1. Demographic information:
Age: Less than 30, (30 – 40), (41-50), or over 50.
Position: Students, administration, or faculty members.
Marital status: Single, married, or divorced.
Do you currently engage in physical activity or sports? Yes No

2. Motivations and challenges scale to physical activity

Motivations Part	Items
Social motives	1. Doing physical activity helps me to form social relationships.<br>2. Being physically active helps me to make new friends.<br>3. I belong to a social group (friends/colleagues/family) that encourages me to be physically active.<br>4. I think that physical activity is a social activity, and that's why I want to do it.
Psychological motives	1. Physical activity can help me to lower stress and the pressures of daily life (study/work).<br>2. I think that doing physical activity helps me to relax and to break out of the daily routine.<br>3. Doing physical exercise helps me to build my self-confidence.<br>4. Doing physical activity helps me to control my emotions and makes me feel good about myself.
Healthy motives	1. I feel healthy when I do physical activity<br>2. Being physically active helps me maintain healthy habits in my daily routine.<br>3. Practicing physical activity contributes to the prevention of joint and muscle pain that results from physical inactivity.<br>4. Being physically active helps me maintain my youth and delays the symptoms of premature aging.<br>5. I do physical activity because the doctor asked me to do it.<br>6. Doing physical activity helps to prevent chronic diseases and heart disease.
fitness related motives	I do physical activity to lose weight.<br>2. I do physical activity to keep my body fit and to have a fit appearance.<br>3. do physical activity to improve the flexibility of my body's muscles and joints.<br>4. I do physical activity to maintain an ideal body weight.
Athletic-inclination motives	1. I have been fascinated by physical activity since childhood.<br>2. I love to do physical activity.<br>3. Physically active helps me to learn new skills.

Challenge's part	Items
Professional or educational	1. Exercising in physical activity may negatively affect my work/studies.<br>2. My work/study requires a great deal of effort and does not leave for me time for physical activity.<br>3. I don't have enough time to do any physical activity due to work/study pressures.
Financial and services	1. Doing physical activity at health clubs is very expensive.<br>2. I can't buy gym equipment for home exercise<br>3. The sport services offered on campus are low quality.<br>4. The university's programs available for physical activity are insufficient.<br>5. There is no health club in the residential neighborhood where I live.<br>6. There are no public facilities for physical activity (health walkway, garden, etc) close to my place of residence.
Health challenges	1. I have a physical injury that prevents me from doing physical activity.<br>2. I suffer from chronic diseases that prevent me from doing physical activity.<br>3. I think that I don't have the physical fitness to do physical activity.<br>4. I am in good health and do not need to do physical activity.
Psychological obstacles	1. I have a fear of being injured during physical activity.<br>2. I am embarrassed to do physical activity at public facilities.<br>3. I feel ashamed of my body and the way others see me.
Societal/family obstacles	1. My age does not allow me to be involved with sports/physical activity.<br>2. No one encourages me to do physical activity.<br>3. I don't have enough time for physical activity because of my family obligations.<br>4. My social surroundings (friends/colleagues/family) do not engage in physical activity.<br>5. I don't have the social skills to participate in a group and do physical activity.
The absence of awareness	1. I am not interested in physical activity/sports.<br>2. Physical activities are too stressful.<br>3. I don't know enough about the benefits of physical activity
